# Fatal Administration of Propofol: A Comprehensive Narrative Forensic Review of Propofol Deaths From Accidents, Suicides, and Homicides

**DOI:** 10.7759/cureus.111853

**Published:** 2026-06-30

**Authors:** Philip R Cohen

**Affiliations:** 1 Dermatology, University of California Davis Medical Center, Sacramento, USA; 2 Dermatology, Touro University California College of Osteopathic Medicine, Vallejo, USA

**Keywords:** accidental, dermatology, drug, forensic, homicide, injection, murder, needle, propofol, suicide

## Abstract

Accidental death, suicide, and homicide have resulted from the intravenous injection of propofol. There have been at least 30 accidental victims of propofol self-administration; more than 75% were medical workers who chronically used it as a recreational drug. At least 13 of the 14 individuals who committed suicide by injecting themselves with propofol were either a physician or a nurse. A fatal injection of propofol was used to murder a minimum of 10 people; for many of these deceased persons, neither the drug nor homicide was initially suspected at the time of death. Therefore, the discovery of an intravenous needle mark wound, or additional clinical history, may lead to further evaluation of the death scene or the victim or both and result in the appropriate toxicology studies of the deceased person, which can establish that the cause of death was from propofol and the manner of death was homicide. A meticulous and thorough examination of the cutaneous and mucosal surfaces of a dead person may provide the essential forensic dermatology clue to either the cause of death (which is the specific illness, pathophysiologic aberration, or trauma that results in the individual's dying) or the manner of death (which is the situation of how the death occurred, such as the consequence to an accident, a suicide, a homicide or a natural sequela of disease, aging or bodily malfunctioning) or both. In summary, propofol intravenous injection can result in accidental death, suicide, or homicide; the number of needle mark wounds can be multiple or solitary. A thorough search of the death scene and a comprehensive forensic examination of the victim may be useful in establishing the manner of death when the cause of death has been attributed to propofol injection. The intent of this paper is to provide a comprehensive narrative forensic review of propofol deaths from accidents, suicides, and homicides.

## Introduction and background

Propofol (2,6-diisopropylphenol) is an anesthetic. It is an intravenous agent with a rapid onset of sedation and unconsciousness. It is potent and short-acting [1-71].

Propofol was initially described in the medical literature in 1977. The drug was dissolved in polyoxyl 35 castor oil, which is a synthetic non-ionic surfactant and solubilizing agent. In this formulation, the drug resulted in anaphylaxis, and it was withdrawn [1-14].

The agent was subsequently reintroduced in the United Kingdom in 1986; in contrast to the original drug, this new formulation of propofol was now dissolved in an oil-in-water lipid emulsion. The modern composition of the propofol accounted for its ‘milk of amnesia’ appearance. In 1989, the agent was approved for use in the United States of America [1-14].

Background

Medication errors during unmonitored procedural sedation, non-intentional overdose, or propofol-related infusion syndrome are all potential causes of operation-related propofol deaths. In addition to propofol deaths associated with operations, fatal delivery of propofol may result from accidental recreational use, self-administration during suicide, or injection by a murderer [1-71].

The first accidental death associated with recreational use of propofol was in 1992. Shortly thereafter, propofol-related suicide was documented in 1994. In 2005, the first murder attributed to propofol was discovered [1-71].

Non-surgical lethal administration of propofol has been reported in at least 54 individuals [5,7-9,15-59]. However, it is likely that this is an underestimate of the number of deaths that have been caused by the drug [25].

Search strategy

This is a narrative review; the PubMed search engine was used to obtain titles and abstracts of relevant articles. The search on PubMed was conducted from 1986 (when the reformulated agent was initially used in the United Kingdom) to 2026. The following terms, by themselves and in combination, were used to screen for appropriate published papers that described not only the features of but also death from propofol: accidental, dermatology, drug, forensic, homicide, injection, murder, needle, propofol, and suicide. The articles obtained from the searches were reviewed, and the relevant references cited after the articles were evaluated.

Since this is a narrative review, quantitative synthesis (meta-analysis) was not undertaken. The paper relies on heterogenous patient case reports. Therefore, the detailed information is appropriately summarized in tables, and the data is not combined statistically.

There are limitations in the study design. Only a single medical search engine (PubMed) was used to initially obtain references; however, all the papers obtained were thoroughly evaluated, and additional appropriate papers discovered in their references cited were obtained for assessment. In addition, duplicate reports on the same cases were included to provide additional details on the individual case; however, in these circumstances, the case was only included once in the appropriate death category, and all the additional papers were only attributed to that specific case. Also, the papers cited were predominantly written in English; however, non-English papers were also included, and they were translated using computer-generated translation into English [1-71].

## Review

Overview

The forensic assessment of a dead person includes a determination of the manner of death. The manner of death is the situation of how the death occurred, such as the consequence to an accident, a suicide, a homicide or a natural sequela of disease, aging or bodily malfunctioning; in some circumstances, the manner of death is undetermined. Propofol intravenous injection can result in accidental death (a fatality that results from an unintended, sudden, and unforeseen external event), suicide (the act of intentionally causing one's own death), or homicide (when an individual causes the death of another person) [1-71].

The characteristics of propofol are described. The manner of death from self-administration of lethal propofol injection can be either accidental (when the drug is being used in a recreational manner) or suicide (when the injection is intended to be fatal). In addition, although less common, the intravenous injection of propofol--either as a single drug or as one of several medications--has been used to murder individuals [5,7-9,15-59].

General features of propofol

The primary indication for propofol is as an anesthetic agent. Propofol has also been effectively used to treat alcohol withdrawal [6,69]. Other uses of the drug include the management of not only migraine headaches but also seizures [70,71]. Currently, propofol is usually restricted by the hospital to individuals with institutional credentials to administer the drug; these may include anesthesiologists, certified registered nurse anesthetists, or critical care physicians (provided they have appropriate training and certification) [1-14].

The most commonly listed therapeutic level for propofol induction of anesthesia is 2.0 to 2.5 milligrams per kilogram; however, in some individuals, anesthesia induction with propofol has been achieved at an even lower range of 1.5 milligrams per kilogram [1-14]. Propofol can be detected in body fluids, such as blood, bile, and urine. It can also be detected not only in solid organs such as the kidney and liver but also in the brain. In addition, a metabolite of the drug (propofol glucuronide) can be demonstrated in hair and nails [7-14,18,24].

Mechanism of propofol

Propofol is a depressant of the central nervous system. The modulation of the gamma-aminobutyric acid A (GABA_A_) receptor in the central nervous system is its primary mechanism of action. By bonding at the interface between the alpha and beta subunits of the post-synaptic GABA_A_ receptor, it enhances the inhibitory effects of gamma-aminobutyric acid [1-14,60-65].

Propofol hyperpolarizes neural membranes by prolonging chloride influx. The duration that the GABA_A_ receptor's chloride channels stay open increases. This results in depressed central nervous system activity and causes unconsciousness, as the hyperpolarization makes it difficult for the neuron to fire an action potential because it cannot reach the necessary threshold [1-14,60-65].

Secondary mechanisms of propofol action include N-methyl-D-aspartate (NMDA) subtype of glutamate receptor blockade. The drug blocks the NMDA receptors and thereby inhibits excitatory glutamatergic activity, which also results in the depressing nervous system stimulation. Other secondary mechanisms of propofol include activation of inhibitory glycine receptors of the brainstem and spinal cord (which results in immobility and muscle relaxation), inhibition of fatty acid amide hydrolase (FAAH), which normally breaks down anandamide (causing increased levels of the endocannabinoid that results in sedation, pain modulation, and antiemetic effects), and modulation of calcium influx [1-14,60-65].

Potential adverse effects of propofol

Propofol has several potential adverse side effects, including death. Neurotoxicity includes decreased cerebral blood flow and cerebral metabolic rate for oxygen. In addition to depressing the central nervous system, propofol can also have adverse effects on the respiratory system (by decreasing both the respiratory rate and tidal volume), the cardiovascular system (by decreasing the blood pressure, which can result in not only hypotension but also bradycardia), and the skeletal system (manifested by myoclonus that occasionally develops in the skeletal muscles) [2,3,66-68].

Propofol-related deaths

Propofol is not a common agent of abuse. People who abuse propofol are usually individuals with a healthcare-related occupation. They typically access the drug at their place of employment [4,13-16,27].

At least 54 individuals have been described in whom death has been associated with propofol administration [5,7-9,15-59]. The manner of death associated with propofol can be either accidental [7-9,15-25], suicide [15,17,19,21,27-31], or homicide [5,31-59]. In the setting of an accidental death or suicide, the person needs to be capable of injecting the drug into their own vein. However, when propofol is used to murder someone, the assailant needs to inject the drug intravenously into the victim.

Accidental propofol deaths

The first published report of death by propofol overdosage was in 1992; the investigator was the assistant director of scientific services at the Victorian Institute of Forensic Pathology in South Melbourne, Victoria, Australia. A 29-year-old woman, a hospital radiographer, accidentally killed herself by self-administering the drug. A total propofol dose of 400 mg was suspected to have been injected. The drug was detected postmortem using high-performance liquid chromatography not only in her blood but also in her urine, muscle, and liver; in addition, propofol was detected in the needle and cannula [21].

At least 30 individuals have been described in whom propofol resulted in an accidental death (Table 1) [7-9,15-25]. Most (76%) of the deceased persons (23 of the 30 people) have been medical workers who chronically used propofol as a recreational drug; indeed, many of them inject themselves frequently, ranging from several times each week to multiple times each day. Their occupation provided them with work-related access to the medication, and they were easily able to secure it [1,10,14,54].

**Table 1:** Propofol fatality caused by accident. C: case; MA: middle aged (usually defined from roughly 40-64 years); mg: milligrams; mL: milliliters; NS: not stated; Ref: references; yrs: years. ^a^Cases 25-30 include six additional cases of accidental propofol deaths that were observed in an email survey of 126 academic anesthesiology training programs (conducted from May 2005 to June 2006), during the prior 10 years. The study had a 100 percent response rate and observed 25 propofol abusers; two of the abusers were concurrently using an opiate drug. The incidence of propofol abuse was 10 per 10,000 anesthesia providers. Seven of the 25 abusers died as a result of propofol abuse; six were anesthesia residents and the other was an operating room anesthesia technician. Given the known history, it appears that the deaths of five of the residents were not suicide-related; no additional comments were provided for the manner of death of the anesthesia technician, but it is assumed to be accidental [15]. ^b^The manner of death was not specifically addressed in the report. Both of the individuals had evidence of prior injury to their skin; the man had vertical scars on his anterior forearm (which may have represented prior sites of intravenous cannulae), and the woman had recent and healed sites of needle punctures on her body. These findings are suggestive of not only prior use but also repeated use of intravenous drugs use and an accidental manner of death [25].

C^a,b^	Age sex	Comments	References
1	19 yrs Woman	A student found dead at her home with a syringe in a small vessel of her right calf. Toxicology detected not only propofol but also chlorpheniramine, diphenhydramine, and fluoxetine in her blood.	[16] C1
2	21 yrs Man	He was a non-medical person who had limited medical knowledge that he acquired by self-study and being a volunteer fire department member; in addition, he had posed as a trained paramedic and emergency physician. He obtained his drugs from pharmacies using stolen and forged prescriptions and had purchased the medical supplies through online auctions from eBay on the internet. He had a venous cannula inserted into his left foot and an empty ampule of propofol (200 mg); he had at least 24 puncture marks on his feet from injecting propofol four or five times each day. Toxicology of his blood revealed not only propofol, but also etomidate and diazepam.	[17]
3	26 yrs Man	A nurse who worked in the intensive care unit. His partner reported that he abused propofol for many years. Three years earlier, he had a history of intravenous drug abuse-related acute renal failure. He had been depressed for six months (and on sick leave from work) and did not abuse propofol during that period. He had returned to work 10 days prior to his death, had no intention to take his own life, and had an appointment for the following day. Toxicology showed both propofol and diphenhydramine.	[9]
4	26 yrs Man	An anesthesiology physician trainee was found unconscious in his bed. His co-workers were aware of his history of propofol abuse. His left forearm was canalized with a butterfly needle attached to an empty syringe. His hands and forearms had multiple needle marks. He was intubated at the scene and admitted to the intensive care unit; his electrocardiogram showed ST-segment elevation in leads V1-V3. Within a half hour, he developed a prolonged QT interval, idiopathic ventricular rhythm, and ventricular fibrillation; cardiopulmonary resuscitation was performed and he died. Toxicology demonstrated not only propofol in his blood and hair but also in the syringe found at the scene.	[18]
5	27 yrs Man	An anesthetic nurse with several puncture wounds suggestive of a history of chronic propofol abuse during the preceding days. He was found with three empty 20 ml propofol ampules (10 mg per ml); this was suggestive of a propofol dose of 600 mg. Unused ampules of propofol were also found in his car.	[19]
6	27 yrs Woman	A nurse found at a hospital dormitory; her forearm vein was canalized by a butterfly needle. Her locker had several empty propofol ampules. She had vital signs when found, was given emergency treatment, and died after 20 hours. Toxicology detected not only propofol but also phentermine, thiopental, and vecuronium in her blood.	[16] C2
7	27 yrs Woman	A doctor was found dead in her studio. An empty ampule of not only propofol but also vecuronium was found in the dustbin. An empty syringe was found beside her body and a syringe filled with white liquid was present in a small vessel at the bend of her arm. Toxicology showed propofol in her blood and vecuronium in her brain tissue.	[16] C4
8	28 yrs Man	A man with one injection mark on the back of his hand was found dead in his room. An empty syringe and empty propofol ampule were found beside his body.	[16] C9
9	28 yrs Woman	A nurse, who stored propofol in her house, died after an injection of propofol.	[16] C15
10	29 yrs Man	An emergency medicine doctor who had a two-year history of propofol addiction, which developed when he repeatedly used the drug for relieving pain caused by nephrolithiasis. Thereafter, he would easily obtain propofol from the hospital and he continued to use the drug for recreational purposes (40-50 mg per injection and approximately 200-300 mg per day). He visited a psychiatric rehabilitation program and was hospitalized after his first evaluation; after 14 days, he refused to participate in therapy and left the hospital. Two years later, the investigators learned that he died from drug intoxication (for which propofol overdose was suspected) and was found dead in his study room in the hospital’s emergency department.	[20]
11	29 yrs Woman	A hospital radiographer who had a history of using propofol at home. She referred to propofol as “the milk of human kindness.” She was found with two empty 20 mL ampules of propofol; this suggested a dose of 400 mg. In addition, benzodiazepines were detected in her urine.	[21]
12	30 yrs Man	A doctor was found dead in a hospital room. He had a needle canalizing his forearm and attached to an infusion bag. Two empty syringes and three empty propofol ampules were found next to his body.	[16] C8
13	37 yrs Man	A man had lots of injection marks. He was found dead in a restroom. An ampule of propofol was found at the scene.	[16] C13
14	38 yrs Woman	An anesthesiologist had a history of propofol abuse. For a few months preceding her death, both her family and her co-worker were aware of her propofol abuse. Three empty 20 mL vials of propofol (which suggested a dose of 600 mg) were found.	[22]
15	42 yrs Woman	A nurse, with two injection marks on her forearm, was found dead in her room. Two empty propofol ampules and two blood-stained syringes were found beside her body.	[16] C3
16	42 yrs Woman	A nurse anesthetist “used propofol every day for the purpose of narcotic.” Next to her body were 14 empty ampules of propofol (200 mg per 20 ml) and two full 20 ml syringes; based on these findings, her dose of propofol was 2000 mg.	[23]
17	44 yrs Woman	A nurse in the anesthesiology department was a known abuser of anesthetic agents for many years. Toxicology screening demonstrated propofol, midazolam, and ethanol.	[8]
18	46 yrs Woman	A woman was found dead in her room. She had one injection mark on the back of her hand. An empty syringe was found beside her body.	[16] C10
19	50 yrs Man	A doctor, with 11 injection marks in his arm, was found dead in an endoscope room. There were 21 blood-stained syringes next to his body. Toxicology screening of his blood demonstrated propofol and ethanol.	[16] C7
20	56 yrs Woman	A propofol addict died after the self-administration of the drug. Toxicology showed not only propofol but also diazepam, fluoxetine, and ketamine, and lidocaine.	[16] C11
21	MA Man	A healthcare provider with unintentional intoxication resulting from propofol and tramadol self-administration. There was neither a suicide note nor any prior suicide attempts; he had no history of psychiatric treatment.	[7]
22	NS Man	A psychiatry resident with a history of depression, a suicide attempt 1.5 years prior (consisting of a massive ingestion of venlafaxine and quetiapine), and chronic abuse of drugs, including propofol. A near-empty 50-mL vial of propofol and a syringe filled with three mL of the drug was present; however, his autopsy revealed multiple drugs in his blood: propofol, fentanyl, several benzodiazepines (nordiazepam, 7-aminoclonazepam, alprazolam), venlafaxine, O-desmethylvenlafaxine, and psychoactive drugs (quetiapine). The investigators stated: “although death by suicide cannot be totally ruled out, the findings of this case suggest an accidental death by … overdose.”	[24]
23	NS^b^ Man	An adult health care professional was discovered unresponsive in his bed. Two intravenous drips (containing a white substance in the tube) were attached to cannulae in the man’s right antecubital fossa and medial aspect of the right ankle. He also had vertical scars over his anterior right forearm. Toxicology studies showed not only propofol and propofol glucuronide (a metabolite of propofol) but also ethanol and midazolam.	[25] C2
24	NS^b^ Woman	An adult health care professional was discovered dead in a room of a healthcare facility where she worked. An intravenous drip was attached to a machine that provides continuous infusion; the drip was connected to an access line in her left inner leg. A second intravenous cannulae was in the dorsum of her left foot. She had a number of minor bruises over the surface of her body; some of the bruises (such as those on the anterior and medial aspects of her right thigh) were associated with recent and healed needle puncture marks. Empty vial holders, syringes, and needles were also found in a backpack in the room. Toxicology studies showed not only propofol glucuronide (a metabolite of propofol) but also cyclizine, diazepam, lignocaine, lorazepam, and midazolam.	[25] C1

Recreational use of propofol was equally common in men (12 of the individuals) and women (12 of the individuals). The gender was not described in six individuals (Table 1) [7-9,15-25].

The exact age was provided for 20 of the victims. They included nine men and 11 women. Their age ranged from 19 years to 56 years; the median age was 29 years (Table 1) [7-9,15-25].

The men ranged in age from 21 years to 50 years. The median age was 28 years. Most of the men (six of nine, 67%) were in their third decade between the ages of 21 and 29 years (Table 1) [7-9,15-25].

The women ranged in age from 19 years to 56 years. The median age was 38 years. Most of the women were either in their third decade (four of 11, 36%) or fifth decade (four of 11, 36%). The younger women ranged from 27 to 29 years, whereas the older women ranged from 42 to 46 years (Table 1) [7-9,15-25].

Nearly half of the 30 individuals (at least 13 of the victims) who accidentally died from self-administration of propofol were concurrently using at least one additional drug or ethanol (Table 1) [7-9,15-25]. Based on the observations of recreational propofol use, hospitals and nations, such as Korea, subsequently placed additional restrictions on workers' access to the drug [13,15,16].

Suicidal propofol deaths

Within two years of the initial report of accidental death by propofol overdosage by self-administration, the first person who died from suicide by self-injection of propofol was published in 1994; the researchers were from the Institute of Science and Forensic Medicine at the National Blood Center in Singapore. A 37-year-old Chinese male medical doctor who was employed at one of the Government hospitals injected himself with 1600 mg of propofol; he was upset after his mistress, with whom he was having an extramarital affair, ended their relationship. He was found dead in a room that he had rented. He had hypodermic needles inserted into his dorsal left hand (two needles) and right hand (one needle); each of the needles was attached to a drip. At the scene, there were eight empty propofol ampules (200 mg per ampule), six broken calcium gluconate ampules (each stained with a milky liquid), and five broken potassium chloride ampules (each stained with a clear liquid) [29].

There is a minimum of 14 people who committed suicide by injecting themselves with propofol (Table 2) [15,17,19,21,26-31]. However, two of the women hanged themselves after they had they had self-administered the propofol [16]. At least 13 of the 14 individuals were either physicians or nurses. Like the victims whose manner of death was accidental, all these individuals had an occupation that allowed them access to the propofol at their place of employment (Table 2) [15,17,19,21,26-31].

**Table 2:** Propofol fatality caused by suicide. C: case; mg: milligrams; mL: milliliters; Refs: references; yrs: years ^a^The abstract in a case of “a fatality due to propofol poisoning” stated that the report describes a suicide by self-administration of propofol in a 29-year-old woman radiographer. However, the final paragraph of the paper stated that “the coroner returned the finding that the deceased died from an overdose of propofol and that there was no indication that she intended to take her own life or that any second party was involved [21].” This case was included in Table 1. ^b^Case 14 is a case of suicidal propofol death that was observed in an email survey of 126 academic anesthesiology training programs (conducted from May 2005 to June 2006) during the prior 10 years; the study observed 25 propofol abusers. Given the known history, it appears that the manner of death of one of the residents was suicide, as the individual had had previous suicide attempts [15]. ^c^Propofol was detected in 131 cases (0.88%) out of 14,673 autopsied cases within six years (2005 to 2010) at the National Forensic Service, Korea. In three of the women, the manner of death was suggestive of suicide: two of the women died from hanging themselves, and the investigators emphasized that one of the women suffered from depression [19]. ^d^The State Institute of Legal and Social Medicine in Berlin (Berlin, Germany), from 1998 to 2011, identified six deaths (of a total of 15,300 autopsies) resulting from self-administration of propofol; all were health care professionals and suicides [27]. Medical supplies for injection were found still on, or near, the bodies at the scene [27,28].

C^a,b^	Age sex	Comments	References
1	28 yrs^c^ Woman	A woman with injection marks that were found on her arm. She hanged herself.	[19] C12
2	29 yrs Man	An anesthesiologist’s fiancée had left him. He was found dead in the living room of the apartment where they used to live together. Inserted into his left antecubital fossa was an intravenous infusion set attached to a saline solution drip, which contained a white residual liquid; another nearly empty similar solution was present. In conclusion, based on the evidence at the scene, this was an intentional suicide.	[26]
3	33 yrs^c^ Woman	A nurse was found dead after an intravenous injection. She had suffered from depression.	[19] C14
4	34 yrs^c^ Woman	A nurse, after an injection of propofol, hanged herself. At the scene, some hypnotic drugs were found. In addition to propofol, toxicology showed alprazolam, clotiazepam, fluoxetine, triazolam, and quetiapine in her blood.	[37] C16
5	36 yrs^d^ Man	An intensive care unit nurse had an infusion catheter inserted in a vein in his left antecubital fossa; the catheter was attached to an infusion bottle (500 mL) containing a little milky-colored fluid. He was found dead in his apartment. A sentence was written on the infusion bottle that implied his suicide. In addition to propofol, toxicology studies of his blood showed diazepam and midazolam.	[27] C3
6	37 yrs Man	A Government hospital medical doctor was having an extra-marital affair. He was upset after being told by his mistress to break off the relationship. His was found dead in a rented room with two hypodermic needles inserted into his left hand and eight empty propofol ampules (200 mg propofol per ampule); therefore, the estimated dose of propofol was 1600 mg. Therefore, suicide by propofol overdose was the verdict of the coroner.	[29]
7	42 yrs^d^ Woman	An anesthesiologist had an infusion catheter inserted into the vein of her right forearm. The catheter was attached to an infusion bottle (50 mL) of propofol containing a milky-colored fluid. She was found dead in a night-duty room in the hospital. The anterior surface of both of her forearms had multiple fresh puncture marks. In addition to propofol, toxicology studies of her blood showed amiodarone.	[27] C4
8	46 yrs^d^ Man	An intensive care unit nurse, with multiple fresh puncture marks on the back of both hands, was found dead in his apartment. Near his body, not only five unopened bottles of propofol but also two partly empty infusion bottles and two used injection syringes were found. In addition, a suicide note was found. In addition to propofol, toxicology studies of his blood showed codeine, diazepam, morphine, piritramide, and tramadol.	[27] C6
9	47 yrs^d^ Man	An anesthesiologist was found dead in his apartment. He had an infusion catheter inserted in a vein in his left antecubital fossa; the catheter was attached to an infusion bottle containing a milky-colored fluid. There was a suicide note; the note indicated that the man had committed suicide due to grief from the death of his partner. In addition to propofol, toxicology studies of his blood showed diazepam.	[27] C2
10	48 yrs^d^ Man	A general practitioner (medical doctor) was found dead in his own clinic. An infusion catheter, attached to an infusion bottle containing a light milky-colored fluid, was inserted into a vein in his left antecubital fossa. He had previous suicide attempts and the financial difficulty of the clinic was a considerable burden to him. In addition to propofol, toxicology studies of his blood showed alfentanil and ethanol.	[27] C1
11	50 yrs Man	An anesthesiologist was found dead by his brother in the house where he lived alone since separated from his wife. A suicide note was found on the living room table. There was pornographic material on his computer and in his closet in two knapsacks; the latter also contained four plastic phalluses. In addition to propofol, toxicology showed amitriptyline, ethanol, ketoprofen, midazolam, thiopental, and zolpidem in his blood.	[30]
12	54 yrs^d^ Woman	An anesthesiologist, with multiple fresh puncture marks on the back of her right hand, anterior surface of the left wrist, and on the back of both feet was found dead in her apartment. Two used injection syringes and an empty bottle (50 mL) of propofol were found next to her body. The woman not only had a long-term history of depressive disorder but also previous suicide attempts. In addition to propofol, toxicology showed mirtazapine.	[27] C5
13	55 yrs Man	A nurse who was found dead in a hospital operating room with a single acupuncture site on his right ankle and a syringe nearby. The investigators concluded that the “nurse committed suicide with an intravenous infusion of propofol.”	[31] C2

Self-administration of propofol to kill oneself occurred almost twice as often in men (eight) than in women (five); the gender was not described in one individual. The exact age was provided for 13 of the decedents. Their age ranged from 28 years to 55 years; the median age was 42 years (Table 2) [15,17,19,21,26-31].

The men ranged in age from 29 years to 55 years. The median age was 47 years. Most of the men (three of eight) were in their fifth decade between the ages of 46 and 48 years (Table 2) [15,17,19,21,26-31].

The women ranged in age from 28 years to 54 years. The median age was 34 years. Most of the women (two of five) were either in their fourth decade and were either 33 or 34 years old (Table 2) [15,17,19,21,26-31].

At least eight of the 14 decedents who died from suicide following a lethal self-injection of propofol were concurrently using at least one additional drug or ethanol (Table 2) [15,17,19,21,26-31]. One of the women was also taking amiodarone, which was detected on the toxicology studies [27].

Homicide propofol deaths

In addition to accidental deaths and suicides, propofol has been documented as a drug used to murder a minimum of 10 people (Table 3) [5,31-59]. The first documented case of murder resulting from propofol as the causative agent occurred in 2005. The details of the woman’s death are summarized in Appendix 1 [35,46-59].

**Table 3:** Propofol fatality caused by murder. C: case; mg: milligrams; mL: milliliters; Refs: references; yrs: years. ^a^Propofol was detected in 131 cases (0.88%) out of 14,673 autopsied cases within six years (2005 to 2010) at the National Forensic Service, Korea. In 16 autopsied cases among the 131 autopsied cases, the investigators determined that the decedent’s fatality resulted from an accidental death after self-administration of propofol. In this woman, the police did not find any evidence in the room with the nude woman. The circumstances of the death scene were very suspicious for the manner of death to be attributed to murder [16]. ^b^Cases 7-10 all involved deaths (occurring from 2014 to 2019) of elderly individuals with comorbid medical conditions who experienced an acute problem at their residence and died within 15 to 40 minutes after emergency medical therapy that was coordinated by the same medical doctor; eyewitnesses claimed that the victims died shortly after the doctor injected a milky solution. The bodies were exhumed in 2019; toxicology studies showed not only propofol (in all four cases) but also diazepam (Case 10) and/or morphine (Cases 7, 9, and 10) [45].

C	Age sex	Comments	References
1	24 yrs Woman	A college student (Michelle Herndon) was injected with 400 mg of propofol into her left arm vein by a male nurse (Oliver O’Quinn). Her toxicology also demonstrated promethazine in her blood. A paper published in the medical literature, describing some of the circumstances of the decedent, was written by Robert R. Kirby, MD (who in 2005 was a Professor and Chairman of the Anesthesia Department at the University of Florida and was an Emeritus Professor in 2009 when the report was published), James M. Colaw, JD (who was one of the prosecuting attorneys with the Eighth Judicial Circuit of Florida in Gainesville), and Michael M. Douglas, BA in Criminal Justice (who was a Sergeant in the Tactical Impact Unit of the Gainesville Police Department) [54].	[35,46-59]
2	38 yrs^a^ Woman	The woman was found dead and naked in a hotel room. The police could not find any evidence (such as propofol ampules or needles or syringes) in the room. Toxicology studies showed propofol in the blood.	[16] C6
3	41 yrs Man	A man presented to the hospital with acute gastritis and diarrhea; it was subsequently discovered that his companion (who had been a surgeon for four years, who hated the victim, and who had studied in the anesthesia department and had anesthetic knowledge) had added mannitol to the victim’s drink, which caused the diarrhea. The man was hospitalized and treated with therapeutic fasting (consisting of abstinence of all oral liquids and foods), intravenous fluids, and antibiotics (levofloxan, gentamycin, and clindamycin). Within two minutes after a transfusion, the victim developed shortness of breath, cyanosis, and died from respiratory depression. His murder had been disguised as a medical accident; the companion surgeon falsely claimed to be the decedent’s brother and demanded monetary compensation from the hospital. Eventually the surgeon confessed to not only prompting the victim to drink large quantities of the mannitol aqueous solution but also injecting him with midazolam (10 mg), propofol (400 mg), and vecuronium (eight mg) which were all of detected from the victim’s heart, blood, liver, kidney, and urine during the postmortem toxicology studies.	[32,33]
4	41 yrs Woman	Two female nurses (a mother and daughter) decided to commit suicide with propofol that the daughter had stolen from the hospital where she worked. The mother injected the propofol into her daughter; the mother then changed her mind and did not kill herself. The daughter was found dead sitting on a chair near a bed in a hotel room; there were empty vials of propofol (four 10 mg/mL vials and two 20 mg/mL vials), syringes and needles. The victim’s left elbow, forearm, hand and foot (ankle) had signs of needle punctures.	[31] C1
5	50 yrs Man	Michael Jackson (the “King of Pop”)was injected with only 25 mg of propofol by Dr. Conrad Murray (his personal physician) on June, 2009 for insomnia. Dr. Murray was convicted of involuntary manslaughter, his medical license was revoked, and he was sentenced to four years in prison; he was released after two years because of good behavior and due to overcrowding of the California prisons.	[5,34-38]
6	65 yrs Man	Billy Junior Shaw was killed by his 32-year-old stepdaughter Karri Denise Willoughby on April, 2008. She was a registered nurse in Chattanooga, Alabama. The initial report suggested that he died of a myocardial infarction. His body was exhumed one year later and the autopsy showed an elevated level of propofol. The toxicology study proved that he died from a lethal injection of propofol and his cause of death was revised from natural to homicide. Ms. Willoughby pleaded guilty to killing Mr. Shaw and was sentenced to 20 years in prison.	[35,39-44]
7	73 yrs^b^ Man	He had metastatic colon cancer (to the liver and lung), and first degree heart block. He had a respiratory crisis in the nursing home where he lived. The doctor decided to sedate him; he developed a respiratory blockage and died shortly thereafter.	[45] C3
8	81 yrs^b^ Woman	She had type II diabetes and a meningioma. She was found in a state of coma and gasping; a hemorrhagic stroke from a hypertensive crisis was suspected. The doctor arrived and hospitalization was not suggested; the family was not aware of her receiving palliative therapy. Death was confirmed shortly after the doctor arrived.	[45] C4
9	84 yrs^b^ Woman	She had a history of myocardial infarction. She had a syncopal episode at home, and was found by the doctor to be in cardiopulmonary peri-arrest and died.	[45] C1
10	92 yrs^b^ Woman	She had a history of heart disease and hypertension. Medical services were called to the home to evaluate an illness. The doctor arrived and she was in shock and gasping. Terminal septic shock was diagnosed, circulatory support was initiated, and she died.	[45] C2

A health care worker was responsible for the killing at least nine of the people; six of the victims were killed by a physician (Table 3) [5,31-59]. An Italian medical doctor was responsible for the fatal injections of four elderly individuals [45]. One man was killed by a Chinese surgeon who hated his male companion; after the surgeon planned and executed the murder, he unsuccessfully requested financial compensation from the hospital [32,33]. Dr. Conrad Murray, who was found guilty of involuntary manslaughter, was responsible for the death of the singer Michael Jackson [5,34-38].

Three of the decedents were killed by a nurse (Table 3) [5,31-59]. Michelle Herndon was killed by a man she had befriended (Oliver O’Quinn, a surgical intensive care nurse) [35,46-59]. Billy Shaw was killed by his stepdaughter, Karri Willoughby, who was a registered nurse and worked in Chattanooga, Alabama [35,39-44]. A daughter (who was a nurse and was the person who had obtained the propofol) was killed by her mother (who was also a nurse); the two women had planned a double suicide; however, after the mother had assisted with her daughter’s death, she decided that she wanted to live and did not kill herself [31].

The manner of death is speculated to be homicide in a woman who was discovered naked and dead in a hotel room; her toxicology studies documented propofol in her blood. During the investigation of the death scene, the police did not find any evidence. The absence of any drug-related items, such as needles and syringes, tubing or bottles for intravenous administration, or propofol ampules, in the room with the decedent is unexpected and was not observed in any of the victims who had an accidental or suicide death from self-administration of propofol. Therefore, it is reasonable to speculate that the circumstances associated with this woman’s death are extremely suspicious that the victim may have been murdered by someone who removed all of these items [16].

Propofol requires intravenous administration. In all situations of murder, the victim had to consensually or involuntarily permit the assailant to inject the drug. The 24-year-old woman who suffered from debilitating migraine headaches trusted her assailant to be treating her with a therapeutic agent to resolve her pain. The daughter (who had made a suicide pact with her mother) trusted her mother to be assisting her to fulfill their intended plan. Michael Jackson trusted his physician to be helping him to sleep. The elderly patients trusted the doctor who came to assist them and allowed for the intravenous line to be established for their care. The hospitalized man with mannitol-induced gastritis already had an intravenous line established, and the assailant injected the drugs while the victim was asleep. It was not described how Billy Shaw’s stepdaughter convinced him to allow her to inject the drug (Table 3) [5,31-59].

The incidence of propofol-associated murders may be greater than those cases described in the literature. The possibility of an intravenous drug being related to the death of the 24-year-old college student was suggested after the meticulous examination of her body by the assistant medical examiner; the observation of a needle puncture site prompted investigators to examine both the death scene and the surrounding area for the needles and syringes associated with her antecubital intravenous needle puncture wound mark. This search led to the discovery of the propofol vials, which prompted the toxicologist to include the drug in the evaluation of the woman’s blood [35,46-59].

However, injection of an intravenously administered drug was not initially suspected in several of the victims whose deaths were related to propofol. One woman was found nude, and there was no drug-related evidence at the scene; the cause of her death was determined after toxicology studies demonstrated propofol in her blood [16]. In addition, the cause and manner of death were initially misinterpreted in half (five) of the victims; the correct manner of death was only established after the bodies were exhumed and toxicology studies were performed that discovered the presence of propofol [35,39-44]. Therefore, it is possible that additional murders related to propofol injection may have occurred and were not suspected or discovered.

## Conclusions

Fatal intravenous injection of propofol has been demonstrated in accidental deaths, suicides, and homicides. More than three-quarters of at least 30 accidental victims of propofol self-administration have been medical workers who chronically used it as a recreational drug. At least 14 individuals have injected themselves with propofol to commit suicide; a minimum of 13 of the deceased persons were either a physician or a nurse. Not less than 10 people have been identified who were murdered after they received a fatal injection of propofol; for many of the dead people, neither the drug nor homicides were initially suspected at the time of death. Subsequent evaluation, prompted by additional clinical history or the discovery of a single intravenous needle mark wound, lead to further examination of the death scene and toxicology studies, which established that the cause of death was from propofol and the manner of death was homicide. Therefore, meticulous and thorough examination of the skin and the mucosa of a dead person may provide a crucial forensic dermatology clue to either the cause of death or the manner of death or both. When the person performing the autopsy conducts a comprehensive cutaneous examination of the dead person and discovers an intravenous injection site, this forensic clue can result in the consideration of the possibility of an unsuspected homicide in the victim. In summary, individuals whose death is associated with propofol have at least one intravenous needle mark wound at the injection site of the drug. Recreational users of propofol typically have numerous injection site wounds. People who have used propofol to commit suicide can have a variable number of additional needle marks. Individuals who have been murdered with propofol usually only have a single wound from the injection site of the drug. Therefore, a comprehensive forensic examination of the dead person during the autopsy may prompt the investigators to consider additional evaluation of the death scene and expand the types of drugs that are evaluated during the toxicology studies when an intravenous needle mark wound is discovered.

## Figures and Tables

**Figure 1:**
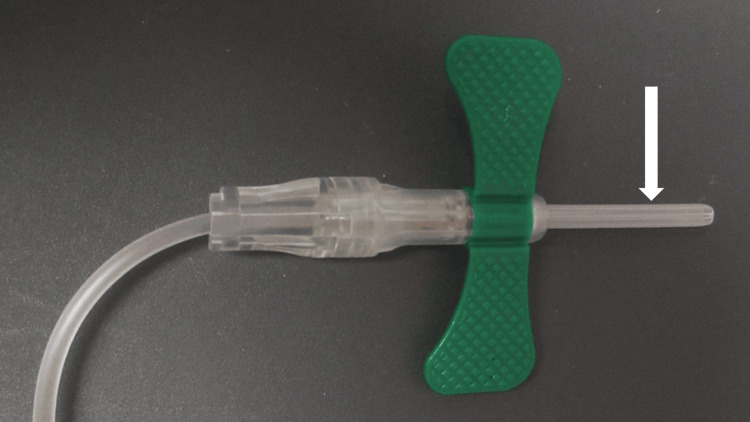
Venipuncture was performed using a butterfly needle. A capped 21-gauge butterfly needle is covered by a plastic needle cap (white arrow). The murderer not only used his teeth to remove the plastic cap that was covering the needle, but also kept the needle cap in his mouth while he was performing the propofol injection. The investigators were able to isolate the killer’s deoxyribonucleic acid (DNA) from his saliva that was found on the needle cap.

## Appendices

Appendix 1

The comprehensive forensic examination that was performed by an assistant medical examiner resulted in the sequence of events that lead to the discovery of the first person who was murdered by propofol injection. The forensic pathologist’s observation of a single needle puncture was crucial to establishing that the death of the dead person resulted from an intravenous injection of the causative drug. In addition, her findings during the examination of the dead woman’s skin prompted the investigators to return to the death scene; the additional investigation by the investigators resulted in the discovery of empty propofol vials and eventually to the murderer’s identity [35,46-59].

A summary of the details, based on the interpretation of a published report in the medical literature and open-access available documents posted on the Internet, is provided. The review of these documents provides the reader with a more complete conceptualization of the victim’s death and the process of discovery of the cause of death and conviction of her killer [35,46-59]. The victim was Michelle Herndon; she died on November 8, 2005. The man who murdered her by injecting her with a fatal dose of propofol, Oliver Travis O’Quinn, was sentenced to life in prison without parole.

Herndon was born on July 15, 1981. She was 24 years old when she was killed. She was in her senior year of college at the University of Florida at Gainesville.

One of Herndon’s friends was Jessica Seipel. Seipel is sharing a house with a man. O’Quinn was her housemate; they had a platonic relationship.

Herndon was friendly towards O’Quinn. However, Herndon and her long-distance ‘on and off’ boyfriend of four years, Jason Dearing, had decided to move forward in their relationship. This upset O’Quinn, who was infatuated with Herndon.

Michelle had a history of migraine headaches. These were very severe when they occurred. She had mentioned them to O’Quinn. At some time, O’Quinn had mentioned that he could provide her with a treatment that would relieve the headache pain.

Herndon and her mother typically spoke to each other at least once daily. Herndon’s last call to her mother was between 10:30 p.m. and 11:30 p.m. on Monday, November 7, 2005. She mentioned that she was suffering from a migraine headache and that O’Quinn would be coming over later that evening to give her something for the pain.

O’Quinn was born on August 18, 1978. He was 27 years old in 2005 when he killed Herndon. When Herndon’s death occurred, O’Quinn was a registered surgical intensive care nurse at Shands Hospital; he had training to perform injections. O’Quinn obtained the propofol from Shands Hospital on November 3, 2005.

O’Quinn arrived either very late in the evening of November 7, 2005, or very early in the morning of Tuesday, November 8, 2005, at Herndon’s home. During the visit, Herndon was experiencing a migraine headache. O’Quinn explained to Herndon that he needed to inject her with the medicine; during the early hours of November 8, 2005, O’Quinn injected Herndon with propofol.

O’Quinn had removed the plastic cap from the butterfly needle with his teeth and held the cap in his mouth during the injection (Figure 1). The saliva found on the plastic needle cap was subsequently tested for deoxyribonucleic acid (DNA); eventually, the test result was received and it showed that the saliva on the plastic needle cap matched O’Quinn’s DNA.

After Herndon died, O’Quinn removed the butterfly needle and put it (along with the plastic cap to the needle, a syringe that contained Herndon’s blood on the inside of the syringe, and two empty propofol vials) into a bag from Publix that lined the waste container in the bathroom. He positioned Herndon’s body face down in her bed to appear like she was sleeping and tucked her in with the covers. He took the Publix bag from the waste container in the bathroom with him as he left the house and locked the front door; he discarded the Publix bag either in or adjacent to a nearby trash can outside the house.

Herndon’s boyfriend, Dearing, had not been able to reach her for nearly two days. Late Wednesday evening (November 9, 2005), at approximately 10:30 p.m. He drove 350 miles and arrived at Herndon’s house shortly after 3 a.m. on Thursday, November 10, 2005.

Dearing realized that something was wrong. The doors to the house were locked, and he saw Herndon through a window; she was lying unresponsive in her bed. He called Michelle’s Herndon’s mother, Belinda, and then he called the police.

Herndon’s body was found in the home she was renting on November 10, 2005; she was 5’5” and 138 pounds (62.6 kg). The police noticed that she was dead and lying face down on her chest and abdomen against the mattress of her bed. O’Quinn had posed her body to look like she was sleeping and tucked her in with the covers pulled up; he locked the front door as he left the house.

The death scene was peaceful, and there was no indication of a struggle or trauma. All the rooms in the home were intact, and none had been ransacked. Herdon’s body did not show any lacerations or bruising, and there was no blood.

The investigators were not able to establish either the cause or the manner of death after their evaluation of the death scene. During the initial inspection of Herndon’s body, the crime scene investigators assumed that she had died from a drug overdose. Detective Michael Douglas of the Gainesville police had not seen any sign of trauma or blood. Detective Douglas initially considered the possibility that the manner of death was suicide and that Herndon had done this to herself by intentionally ingesting a lethal quantity of one more medications.

Detective Douglas then postulated that Herndon may have been a recreational drug user and had overdosed by accident. However, inspection of the house did not reveal any drugs or alcohol. Also, Herdon’s mother strongly emphasized that her daughter had never used drugs. Hence, the possibility of suicide or a drug overdose had been diminished.

There were no signs at the death scene of an intruder. Consideration of a natural manner of death, such as a ruptured aneurysm, was also entertained. As part of procedural protocol, Herndon’s body was sent to the Office of the Medical Examiner (of Florica District 8) for an autopsy.

Dr. Martha Burt, the assistant medical examiner, performed the autopsy. Dr. Burt observed that the dependent areas of Herndon’s body were erythematous and did not blanch; therefore, livor mortis was fixed. Hence, based on this observation, Herndon had been dead more than 12 hours; actually, she had been dead for more than 24 hours and closer to 48 hours.

Dr. Burt suspected that Herndon had been moved and repositioned after her death. When discussing the features of the dead woman’s postmortem hypostasis with Detective Douglas, Dr. Burt stated that Herndon’s pattern of lividity indicated that she had been placed face down, with her face in her pillow, soon after she died. Based on her clinical experience, Dr. Burt mentioned that this was not the way Herndon would land if she had fallen ill and collapsed. Dr. Burt subsequently commented that “If somebody is sick and in the process of dying, most folks tend to end up either on their side or on their back.”

Dr. Burt continued her meticulous evaluation of Herndon’s body. During Dr. Burt’s detailed gross examination, she made a crucial discovery. This observation led to the determination of the cause and manner of Herndon’s death.

During the careful and thorough inspection of Herndon’s skin, Dr. Burt observed a needle puncture wound in Herndon’s left antecubital fossa. The mark from the needle was smaller than a freckle. The postmortem dependent settling of Herndon’s blood, her changes caused by livor mortis, partially hid the tiny puncture.

Dr. Burt contacted Detective Douglas, saying, “I found a needle pucture in Michelle Herndon’s arm. In her left arm. And, in my medical opinion, it appears to be administered by someone with a level of skill.” She emphasized to Detective Douglas that Herndon only had a single needle puncture and that there were no other needle marks, excluding the possibility of Herndon being a closet junkie.

Herdon’s mother mentioned that her daughter was afraid of needles. She also commented that her daughter would not donated blood or sell her plasma. In addition, Crime Scene Investigator Woodmansee stated that, “The medical examiner thought that this injury was done by somebody with medical training because there was no redness or bruising around the site.”

After Dr. Burt shared her findings with Detective Douglas, both Detective Douglas and Crime Scene Investigator Woodmansee returned to the scene and expanded their search. They noted a clear plastic grocery bag from Publix that was located adjacent to an outdoor trash barrel beside the house Herndon was renting. It was a stray garbage bag that had inadvertently been left behind when the trash was collected earlier that day. In the house, they noted that the bathroom trash was missing; specifically, the trash can in the bathroom was not only empty but also there was no bag within the bathroom waste container to collect trash.

The bag from Publix was tied shut. The bag contained mail addressed to Herndon. It also contained a pediatric butterfly needle along with its plastic cap, two empty vials of propofol, and a syringe (with blood staining on its inside surface). Subsequent studies of the blood within the syringe matched Herndon’s DNA.

In addition, small vials of midazolam and etomidate were also noted inside the Publix bag. Although these medications were present in the bag, they were not detected in the subsequently performed toxicology studies of Herndon’s blood. The investigators, after assessing the evidence, realized that Herndon could not have self-administered the propofol and thrown out the trash; therefore, they needed to locate a person who had access to the drug and had the knowledge to use it.

Propofol was not usually included in the drugs that were evaluated for during routine postmortem toxicology testing. Based on the discovery of the empty propofol vials, an evaluation for propofol was included in Herndon’s tests. As expected, propofol was discovered in her blood. In addition, the toxicology studies on Herndon’s blood also demonstrated promethazine.

In Dr. Burt’s opinion, the level of propofol found in Herndon’s blood “would require mechanical ventilation to survive.” She eventually testified that the dose of propofol had not only suppressed Herndon’s respiration but also caused extreme sedation. Dr. Burt concluded that Herndon’s death occurred within minutes of the propofol injection.

Each vial of propofol has a unique serial number. The investigators were able to trace the propofol vials to the pharmacy at Shands Hospital. The vials had been located in an Omnicell automated dispensing machine in the intensive care unit.

The hospital kept logs for the automated medication dispensing system; the logs showed that O’Quinn had used his personal access code to withdraw the propofol. Therefore, O’Quinn became the prime suspect. When the DNA results subsequently confirmed that the saliva on the plastic cap of the butterfly needle matched with O’Quinn, he was definitively established as the killer.
